# Epidemiology of Mucormycosis Cases During the Second Wave of COVID-19 in a Tertiary Care Institute in Western Rajasthan, India

**DOI:** 10.7759/cureus.22973

**Published:** 2022-03-08

**Authors:** Arun Kumar, Manoj Verma, Afzal Hakim, Savitri Sharma, Rita Meena, Suman Bhansali

**Affiliations:** 1 Community Medicine, Dr. Sampurnanand (SN) Medical College, Jodhpur, IND

**Keywords:** rhinocerebral mucormycosis, coronavirus disease 2019, covid-19, diabetes, steroid, mucormycosis

## Abstract

Introduction: Mucormycosis is a serious but rare fungal infection that showed a sharp surge during the second wave of coronavirus disease 2019 (COVID-19) in India. This study aimed to describe the epidemiological aspects of mucormycosis cases presenting to a tertiary care centre of Western Rajasthan, India, as well as to identify potential risk factors for Mucormycosis.

Methods: This cross-sectional descriptive study included 55 patients admitted with a diagnosis of mucormycosis between May and June, 2021, covering the second wave's post-COVID-19 period. Data was collected using a pre-designed, semi-structured questionnaire and hospital case sheets.

Results: The mean age of the patients was 54.4±12.53 years, with a male-to-female ratio of 1.89:1. Of the patients, 49% were obese and had no prior history of diabetes. Most COVID-19 patients (54.6%) were treated at home and did not require oxygen support during their COVID-19 course. The majority (89%) were on steroid medication, which was mainly intravenous (93.8%) and lasted 5-10 days in most patients. Rhinoorbitocerebral mucormycosis was the most common type seen in this setting, with symptoms appearing 15-30 days after the onset of COVID-19 symptoms. During the fungal infection, about 61.8% of patients had random blood sugar readings of more than 140 mg/dl. Mortality occurred in 14.5% of patients with mucormycosis. Mortality was observed to be associated with high BMI, raised glycated haemoglobin (HBA1C), and urban residency.

Conclusion: Mucormycosis appears to be caused by impaired glycemic control due to pre-existing or new-onset diabetes, which may be exacerbated by unintentional glucocorticoid use. It is necessary to use steroids with caution and maintain care for at least 15-30 days after the onset of COVID-19 symptoms.

## Introduction

Mucormycosis, or zygomycosis, is a rare disease caused by a filamentous fungus. The nose, paranasal sinuses, and brain are the most common areas affected. It's an opportunistic infection that flourishes in persons who have their immune systems impaired. The causative agents are saprophytic fungus from the Phycomycetes class, order Mucorales, and family Mucoraceae. These fungi include *Mucor*, *Rhizopus*, *Absidia*, *Cunninghamella*, and *Apophysomyces elegans* [1]. Reduced host immunity, as well as conditions like hyperglycemia and iron overload, favour fungal invasion. In immunocompromised patients, the most common route of infection is inhalation of spores from fungi found in soil or organic debris [2]. The fungus develops swiftly and aggressively in those cases, generating a well-defined fulminant and life-threatening disease. To avert long-term neurological disorders and save lives, early intervention is critical. In the majority of instances, it is an acute fungal infection, but chronicity, which is gradual and indolent, has also been recorded [3].

It is said to thrive in hot, humid temperatures and surroundings, especially in tropical locations and throughout the summer. Long-term corticosteroid medication, severe burns, solid organ transplantation, hemochromatosis, HIV, neutropenia, malnutrition, hematologic malignancies, and other disorders are commonly linked [4]. According to earlier research, about 9% of rhinocerebral mucormycosis patients were free of any predisposing factors [5].

The humid environment of the nose and paranasal sinuses fosters the multiplication and invasion of fungi. A unilateral headache behind the eyes and weariness are among the first symptoms. Depending on the duration, host immunity, and severity of the disease, further invasion of the mucosa and bone may occur. Nasal darkening or blackening, blurred or double vision, chest pain, difficulty breathing, and bloody coughing are all symptoms. Bone infiltration is common in cases that go undiagnosed or untreated.CNS involvement causes convulsions, disorientation, impaired mental status, and changes in gait. When the gastrointestinal tract is involved, vomiting and abdominal pain are common [6].

Microscopic examination reveals abnormally wide aseptate fungal hyphae with 90-degree branches, and necrosis and bleeding zones [2]. When it comes to spotting early fungal balls, a CT scan is the method of choice. To be treated, all dead and diseased tissue must be surgically removed. In some cases, this might result in the loss of the upper jaw or even an eye. To cure the infection, a four to six week course of intravenous antifungal medications may be required. One of the most important preventive techniques advised by the Indian Council of Medical Research (ICMR) is diabetes control.

The 2019 novel coronavirus (2019-nCoV) or severe acute respiratory syndrome coronavirus 2 (SARS-CoV-2) started in China and soon spread over the world, becoming a pandemic [7]. COVID-19 illness has been associated with oropharyngeal candidiasis, pneumocystis jiroveci pneumonia, pulmonary aspergillosis, bloodstream candida infections, and other serious opportunistic infections [8,9]. In COVID-19, there have been a few rare case reports of rhino-orbital mucormycosis [10,11]. Steroids, monoclonal antibodies, and broad-spectrum antibiotics used to treat COVID-19 disease have been shown to increase the risk of a new fungal infection or intensify an existing one [10]. Furthermore, COVID-19 patients receiving oxygen therapy in an ICU with a humidifier are susceptible to fungal infection due to moisture exposure. 

Mucormycosis has an incidence rate of 0.005 to 1.7 per million people worldwide [12]. In India, the prevalence is 0.14 per 1000 people, which is almost 80 times greater than in wealthy countries [13]. Mucormycosis has a global mortality rate of 46% [14]. The disease is not new but in the context of the COVID-19 pandemic, particularly during and after the second wave in India, an increase in the number of cases has been seen, which necessitates more investigation. This study was thus conducted with an objective to describe the epidemiological aspects of mucormycosis cases presenting to a tertiary care centre in Western Rajasthan, India, as well as to identify potential risk factors for mucormycosis.

## Materials and methods

The research was carried out at the Department of Community Medicine, Dr. Sampurnanand (S.N.) Medical College in Jodhpur. All the patients of any age or gender who were admitted to Mathura Das Mathur (MDM) Hospital, Jodhpur, India with a confirmed diagnosis of mucormycosis from May to June, 2021, were included in the study. The end of the second wave of COVID-19 across the country as a whole, and in the state, in particular, occurred during this time.

The study was started after approval from the Institutional Ethics committee, Dr. Sampurnanand (S.N.) Medical College, Jodhpur (Approval No. SNMC/IEC/IIP/2021/012). Prior to inclusion in the study, all subjects gave their written informed consent. Data was collected using a pre-designed, semi-structured questionnaire. Patients were asked about their sociodemographic profile, medical history, personal habits, COVID-19-related information, and detailed treatment history during COVID-19 illness if present, including steroid use, diabetes control, mode of oxygen use, hygiene conditions during oxygen use, and current clinical information about mucormycosis. Relevant clinical information was also evaluated on the patient's hospital case sheets.

Statistical analysis

Information collected was entered into a Microsoft Excel spreadsheet (Microsoft Corp., Redmond, Washington). Categorical variables were expressed as frequency and percentage and were analysed using the Chi-Square test or the Fischer Exact test as applicable. Continuous variables are expressed as mean and standard deviation. Histogram was drawn to assess the normality of variables. Independent sample t-test/Mann-Whitney U tests were applied for analysis of continuous variables based on normal approximation. A p-value of < 0.05 was taken as statistically significant. All statistical analyses were done using Epi Info™ version 7.2.1.0 statistical software (Centers for Disease Control and Prevention, Atlanta, Georgia, United States).

## Results

The study comprised a total of 55 mucormycosis patients. The average age of the patients was 54.4±12.53 years. With a male-to-female ratio of 1.89:1, there were more males than females. Of the patients, 49% were obese and the majority (63.7%) had no prior history of diabetes mellitus (Table 1).

**Table 1:** General characteristics of study subjects

		N	%
Age group (in years)	<30	1	1.8
	30-45	9	16.4
	45-60	25	45.5
	≥60	20	36.3
Gender	Male	36	65.5
	Female	19	34.5
Residence	Rural	40	72.7
	Urban	15	27.3
Family type	Nuclear	9	16.4
	Joint	46	83.6
Education status	Illiterate/below secondary	43	78.1
	Secondary and higher	12	21.9
Smoking status	Smoker	6	11.0
BMI (kg/m2)	<18.5	6	11.0
	18.5-24.9	22	40.0
	≥25	27	49.0
Prior diabetic history	Yes	20	36.3
Duration of diabetes (years)	<5	7	35.0
	≥5	13	65.0

In terms of COVID-19 history, the majority of patients (94.5%) had pyrexia as a presenting symptom, with most falling into the moderate-severe group on the CT Severity Scale/Score (CTSS) (Table 2). The Chest CTSS is based on multifocal ground-glass opacities and consolidation as observed in viral pneumonia on high-resolution computed tomography (HRCT) scan commonly in a peripheral distribution [15].

**Table 2:** Distribution of patients according to clinical characteristics of COVID-19 COVID-19: coronavirus disease 2019

		N	%
Symptoms of COVID-19	Fever	52	94.5
	Cough	35	63.6
	Dyspnea	9	16.3
CT Severity Score	<8	7	21.2
	8-15	21	63.6
	>15	5	15.2
	Not done	22	40.0
Minimum SpO2	<90	30	56.6
	90-93	5	9.4
	94-98	18	34.0

More than half of the patients were mostly treated at home in isolation. The majority of patients (54.6%) did not require oxygen support during their COVID-19 treatment and those who did, needed it for 5-10 days. The majority of patients (89%) said they were on steroid medication, which was usually intravenous and lasted 5-10 days (Table 3).

**Table 3:** Treatment characteristics of patients during COVID-19 illness NIV: non-invasive ventilation; COVID-19: coronavirus disease 2019

		N	%
Treatment of COVID-19 primarily at	Home	30	54.5
	Hospital	25	45.5
Oxygen support needed	Yes	25	45.4
	No	30	54.6
Mode of oxygen support	Cylinder	4	16.0
	Line	21	84.0
	Concentrator	2	8.0
	NIV	1	4.0
Days on oxygen support	<5	7	28.0
	5-10	15	60.0
	>10	3	12.0
Oxygen delivery medium	Face mask	14	56.0
	Nasal cannula	4	16.0
	High flow mask	11	44.0
	NIV	2	8.0
Cleaning frequency of oxygen delivery medium	Never	11	44.0
	Daily	5	20.0
	Alternate day	9	36.0
Changing frequency of humidifier	Never	5	20.0
	Daily	8	32.0
	Alternate day	10	40.0
	More than two days	2	8.0
History of steroid intake	Yes	49	89.0
	No	6	11.0
Mode of steroid	Oral only	3	6.1
	IV only	38	77.5
	Both	8	16.4
Duration of IV steroid (days)	<5	10	23.8
	5-10	28	66.6
	>10	4	9.6

Mucormycosis symptoms appeared in the majority of patients (52.7%) within 15-30 days of the onset of COVID-19 symptoms. Headache was the most common presenting symptom (63.7%), followed by facial swelling/numbness (45.5%), and periorbital swelling/redness (41.9%). At the time of mucormycosis, the majority of the patients had abnormal blood sugar and HBA1C levels. The majority of patients (98.1%) were either unvaccinated or only partially vaccinated against COVID-19 (Table 4).

**Table 4:** Distribution of patients according to mucormycosis related clinical characteristics HbA1c: glycated haemoglobin; COVID-19: coronavirus disease 2019

		N	%
Symptoms	Periorbital swelling/redness/dropping of eyelid	23	41.9
	Visual disturbance	10	18.2
	Headache	35	63.7
	Facial swelling/numbness	25	45.5
	Dental pain/jaw pain	16	29.0
	Nasal symptoms(rhinorrhea,epistaxis,black discharge)	7	12.8
Random blood sugar (mg/dl)	<140	21	38.2
	140-200	10	18.2
	>200	24	43.6
HbA1c (%)	<6	21	38.2
	≥6	34	61.8
Symptom onset interval between COVID-19 and mucormycosis (days)	<7	4	7.3
	7-14	10	18.1
	15-30	29	52.7
	>30	12	21.9
COVID-19 vaccination status	Not vaccinated	31	56.3
	Partial vaccinated	23	41.9
	Complete vaccinated	1	1.8
Surgery	Orbital exenteration	3	5.4
	Maxillectomy	16	29.0
	Endoscopic Endonasal Debridement	50	90.9
Outcome	Death	8	14.5
	Survived	47	85.5

A total of 14.5% of patients with treated mucormycosis died. High BMI, abnormal HBA1C, and urban habitation were revealed to be substantially linked with death. According to reports, all of the patients who died were on IV steroid medication at some point in their COVID-19 treatment. Despite the fact that the majority of patients who died had CTSS indicating a history of moderate COVID-19 severity, the link was not found to be significant (Table 5).

**Table 5:** Factors associated with mortality during mucormycosis *Significant at p value<0.05 COVID-19: coronavirus disease 2019; CTSS: computed tomography severity score; HBA1C: glycated haemoglobin; RBS: random blood sugar

		Death	Survived	P-Value
Age (Years) (N=55) Mean±SD		56.75±13.86	54±12.41	0.61
BMI (kg/m2) (Mean±SD) (N=55)		28.71±3.35	24.78±6.27	0.01*
RBS (mg/dl) (Mean±SD) (N=55)		256±114.44	196.06±101.13	0.19
Symptom onset Interval between COVID-19 and mucormycosis (days) (N=55)		21.63±9.88	21.42±11.85	0.95
Residence	Rural (N=40)	3(37.5)	37(78.7)	0.02*
	Urban (N=15)	5(62.5)	10(21.3)	
Prior diabetic history	No (N=35)	3(37.5)	32(68.0)	0.12
	Yes (N=20)	5(62.5)	15(32.0)	
CTSS	<8 (N=07)	1(14.2)	6(23.0)	1.00
	≥8 (N=26)	6(85.8)	20(77.0)	
Oxygen support needed	No (N=28)	5(62.5)	23(49.0)	0.70
	Yes (N=27)	3(37.5)	24(51.0)	
HBA1C (%)	<6 (N=34)	2(25.0)	32(68.0)	0.04*
	≥6 (N=21)	6(75.0)	15(32.0)	
Steroid use	Yes (N=49)	8(100)	41(87.2)	0.57
	No (N=06)	0(0.0)	6(12.8)	
Surgery done	Yes (N=50)	7(87.5)	43(91.4)	0.55
	No (N=05)	1(12.5)	4(8.6)	
Changing frequency of humidifier	Never (N=05)	0(0.0)	5(22.8)	0.67
	Daily (N=08)	2(66.6)	6(27.3)	
	Alternate Day (N=10)	1(33.3)	9(40.9)	
	More than Two Days (N=02)	0(0.0)	2(9.0)	
Cleaning frequency of oxygen delivery medium	Never (N=11)	0(0.0)	11(50.0)	0.27
	Daily (N=05)	1(33.3)	4(18.2)	
	Alternate Day (N=09)	2(66.6)	7(31.8)	

## Discussion

The current investigation included 55 mucormycosis patients who were all found to have a history of either COVID-19 positive status on reverse transcription-polymerase chain reaction (RT-PCR) (43.6%) or COVID-19-like symptoms. The average age of the patients was 54.4 years, with the majority of them being under 45 years old. Males dominated, with a male-to-female ratio of 1.89. The majority of the patients (72.7%) came from rural areas with poor literacy levels. The age and gender distributions are consistent with earlier research. Various investigations found similar findings in terms of age and gender, with the majority of patients being males and a median age of 55-60 years [16,17,18]. Differences in susceptibility and progression of COVID-19, as well as inequities in healthcare access between male and female patients, possibly explain the gender distribution. During the second wave of COVID-19 in India, the age distribution corresponds to the number of patients who were affected in a specific age group [19].

According to published research, COVID-19 is a major antecedent event, and the current study findings support this. The majority of the patients (49%) were overweight or obese. Obesity increases the risk of developing severe COVID-19 and its complications by increasing the incidence of diseases such as renal insufficiency, cardiovascular disease, type 2 diabetes mellitus, certain types of malignancies, and a substantial degree of endothelial dysfunction [20]. The majority of individuals (63.7%) in this study had never experienced diabetes before COVID-19. Those who were diabetic had a long history of diabetes (at least five years). However, previous research has indicated that the majority of individuals have a history of diabetes mellitus [16].

During the mucormycosis period, the majority of patients (61.8%) had HbA1c levels > 6, indicating a lack of long-term glycemic control in conjunction with COVID-19 history. Some prior research [16,21] revealed similar findings of poor glycemic control. COVID-19 infections are more severe in people with diabetes and obesity [22]. As a result, they're more likely to be treated with corticosteroids, which are commonly used to treat COVID-19. However, corticosteroids cause blood glucose levels to drop, especially in people who already have diabetes, increasing the risk of mucormycosis. Furthermore, according to a new study published in Nature, the virus may be causing new-onset diabetes by destroying pancreatic beta cells and aggravating diabetes that already exists [23]. By oxidative and non-oxidative pathways, hyperglycemia caused phagocyte malfunction, decreased chemotaxis, and faulty intracellular killing [24].

During COVID-19 illness, the majority of patients exhibited pyrexia (94.5%), followed by cough (63.6%) and dyspnea (16.3%) as the most common presenting symptoms, though not entirely. This is consistent with previous research [7,10,25]. Of the patients, 60% had CTSS measured by HRCT thorax. The majority (63.6%) of them were in the moderately severe group. Because high CTSS increases the likelihood of problems, most patients who acquired mucormycosis had previous CTSS that might be characterised as moderate to severe.

The majority of patients with mucormycosis (54.5%) received only home isolation treatment. Despite the fact that the majority of patients (56.6%) had a minimum SpO2 of 90% at some point, the majority of such patients (54.6%) were not put on oxygen support. According to a study conducted by the Gujarat state government, the majority of mucormycosis patients did not require oxygen therapy during COVID-19 [19].

Of the patients who had oxygen therapy, 60% required it for 5-10 days. The main oxygen delivery medium among the patients was reported to be face masks, followed by high-flow masks. Cleaning of the oxygen delivery medium was observed to be unpredictable, with 44% of patients reporting that they never cleaned the same thing twice, and humidifier changing was reported to be on alternate days by 40% of patients. According to studies, a completely unclean and filthy method of giving oxygen to patients in many parts of India has resulted in an increase in mucormycosis cases [25]. Reusable oxygen humidifiers may also play a role in the spread of possible nosocomial viruses by producing aerosol particles that reach lung depth following inhalation [26].

Most patients (89%) reported using steroids during COVID-19 treatment, with intravenous steroids (77.5%) being the most common and lasting mostly for 5-10 days (66.6%). Almost identical findings were observed in other studies in which all or the majority of patients were treated with systemic corticosteroids for COVID-19 [17,18]. According to prior research [27], the most commonly used glucocorticoid was parenteral dexamethasone. Steroids are used to treat COVID's side effects rather than the virus itself. It is hazardous and toxic if given early in the viral replication phase [28]. This will lower bodily immunity and allow viruses to replicate more easily. Inadvertent use of steroids without even trying to monitor blood sugar levels, particularly among people with a history of diabetes, predisposes them to an increased risk of COVID severity as well as related comorbidities such as mucormycosis. The widespread usage of glucocorticoids may have led to the increase in COVID-related mucormycosis cases [16,29,30].

The most prevalent presenting symptom of mucormycosis was headache (63.7%), followed by facial swelling and numbness (45.5%) and periorbital swelling and redness/ptosis (45.5% each). Only 12.8% of patients reported nasal issues. However, a previous study indicated that the most prevalent symptoms were vision loss (6/60 in 80.64% of patients) and ophthalmoplegia (77.4%) [18]. Symptoms are often non-specific and vary from person to person. Because the rhinocerebral form was the most common in our area, the symptoms were diverse.

In our study, the majority of patients (52.7%) developed mucormycosis between 15 and 30 days after the onset of COVID-19. Various studies [16,17,27,31] found similar results when symptoms were reported between 15 and 22 days.

One of the most relevant findings in terms of public health was that the vast majority of patients (98.2%) were either unvaccinated or only partially vaccinated. It can be argued that vaccination protects against severe COVID-19 and that, in moderate cases, steroid drugs are not required, reducing the risk of mucormycosis later on.

As per the observations in this study, 14.5% of the patients died. Previous studies have reported greater fatality rates of more than 30% [16,27], but some have also found lower rates of around 9% [18]. Early and successful treatment, as well as a low prevalence of severe, extensive mucormycosis, such as pulmonary involvement, may have contributed to the low mortality among cases in our study.

In terms of factors linked to mortality, a high BMI was discovered to be a major influence. Another significant factor associated with mortality was urban residence, which was likely due to a higher risk of exposure to fungal spores due to unsanitary, overcrowded dwellings, as well as over-the-counter availability and administration of steroids during COVID-19 by paramedical or pharmacy personnel. Deranged high HbA1c has also been found to be significantly linked with mortality; in line with this, an earlier study found that an HbA1c value of 8 was significant in the prediction of survival in patients with mucormycosis [18].

## Conclusions

Deranged glycemic status during COVID-19 disease could be attributed to a preexisting co-morbid state or new-onset diabetes mellitus, which was exacerbated by inadvertent and high-dose use of glucocorticoids, even without hypoxemia, and appears to be one of the most important associated factors with the development of mucormycosis within 15-30 days of the onset of COVID-19 symptoms. Impaired glycemic management has also been linked to mortality. Vaccination appears to protect against a severe form of COVID-19 and, as a result, the development of concurrent fungal infection later. The first 15-30 days after COVID-19 infection appear to be critical, during which patients and clinicians must be watchful and extra cautious for symptoms such as headaches, face swelling, numbness, periorbital symptoms, dental pain, and so on. Treatment that is effective, timely, and appropriate can reduce mortality. Further analytical research investigations are required to determine the components linked to mucormycosis.
